# Emerging Roles of Cyclophilin A in Regulating Viral Cloaking

**DOI:** 10.3389/fmicb.2022.828078

**Published:** 2022-02-15

**Authors:** John E. Mamatis, Isabella E. Pellizzari-Delano, Carla E. Gallardo-Flores, Che C. Colpitts

**Affiliations:** Department of Biomedical and Molecular Sciences, Faculty of Health Sciences, Queen’s University, Kingston, ON, Canada

**Keywords:** virus-host interactions, viral cloaking, RNA viruses, antiviral therapy, cyclophilin A

## Abstract

Cellular cyclophilins (Cyps) such as cyclophilin A (CypA) have emerged as key players at the virus-host interface. As host factors required for the replication of many unrelated viruses, including human immunodeficiency virus (HIV), hepatitis C virus (HCV) and coronaviruses (CoVs), Cyps are attractive targets for antiviral therapy. However, a clear understanding of how these viruses exploit Cyps to promote their replication has yet to be elucidated. Recent findings suggest that CypA contributes to cloaking of viral replication intermediates, an evasion strategy that prevents detection of viral nucleic acid by innate immune sensors. Furthermore, Cyps are emerging to have roles in regulation of cellular antiviral signaling pathways. Recruitment of Cyps by viral proteins may interfere with their ability to regulate these signaling factors. Consistent with disruption of viral cloaking and innate immune evasion, treatment with Cyp inhibitors such as cyclosporine A (CsA) restores antiviral innate immunity and induces expression of a subset of antiviral genes that restrict viral infection, which may help to explain the broad antiviral spectrum of CsA. In this review, we provide an overview of the roles of CypA in viral cloaking and evasion of innate immunity, focusing on the underlying mechanisms and new perspectives for antiviral therapies.

## Introduction

### Antiviral Immunity and Viral Evasion Mechanisms

Successful viral infection depends on evasion of an array of antiviral immune responses. Cells have evolved to sense viral infection *via* recognition of pathogen-associated molecular patterns (PAMPs) by cellular pattern recognition receptors (PRRs), a crucial first step in establishing an effective response to initial viral infection (Kawai and Akira, 2010). Among these PRRs are retinoic acid-inducible gene I (RIG-I), melanoma-differentiation-associated gene 5 (Mda5), and protein kinase R (PKR), which sense viral RNA species (Kato et al., 2006; Sadler and Williams, 2007). RIG-I is activated upon recognition of short double stranded RNA (dsRNA) species as well as 5′ triphosphate RNA (Pichlmair et al., 2006; Kato et al., 2008). RNA binding induces a conformational change in the caspase activation and recruitment domains (CARDs) of RIG-I, allowing interaction with downstream mitochondrial antiviral-signaling protein (MAVS) (Gack et al., 2007). Cyclic GMP-AMP synthase (cGAS) senses cytoplasmic DNA (Sun et al., 2013), leading to the production of cyclic GMP-AMP (cGAMP), which binds to stimulator of interferon genes (STING) (Wu et al., 2013). Ultimately, activation of MAVS or STING results in nuclear translocation of interferon regulatory factor 3 (IRF3) and IRF7, which together with the transcription factor nuclear factor-κB (NF-κB) drive the production of type I interferons (IFNs) that induce expression of antiviral IFN-stimulated genes (ISGs) to restrict viral infection in an autocrine and paracrine manner (Rehwinkel and Gack, 2020). Production of type I IFNs also engages the adaptive immune system to further counteract viral infection.

Cell-autonomous antiviral immune mechanisms also counteract viral replication. For example, PKR binds dsRNA or 5′ triphosphate RNA, resulting in its autophosphorylation and dimerization, allowing phosphorylation of downstream eukaryotic initiation factor 2α (eIF2α) (Nallagatla et al., 2007). Phosphorylated eIF2α plays a critical role in inhibiting protein synthesis to establish an antiviral state (Dever et al., 1992). PKR additionally functions in activating transcription factors such as NF-κB and IRF1 (Bonnet et al., 2000, 2006). Notably, IRF1 has been shown to restrict the replication of a broad range of RNA viruses by directly regulating the expression of a subset of ISGs (Yamane et al., 2019).

Evasion or suppression of type I IFN and cell-autonomous immunity is critical for successful viral replication. Consistently, many viral proteins have evolved to engage with cellular factors to antagonize key signaling pathways involved in antiviral responses. A more passive viral evasion mechanism is the “cloaking” or concealment of viral replication intermediates to prevent their detection by innate immune sensors (Rasaiyaah et al., 2013; Colpitts et al., 2020). Positive-sense RNA viruses achieve this through the rearrangement of intracellular membranes to form cytoplasmic replication organelles (ROs), where RNA replication intermediates are concealed from RNA sensors (e.g., RIG-I, Mda5, PKR). In a mechanistically distinct but conceptually analogous manner, the genetic material of human immunodeficiency virus-1 (HIV-1) remains encapsidated during reverse transcription (Jacques et al., 2016), thus concealing newly synthesized HIV-1 DNA from cytoplasmic DNA sensors (e.g., cGAS). In both cases, interactions with cellular factors are critical for successful cloaking and evasion of innate immunity. Here, we review the roles of cellular cyclophilins (Cyps) in viral cloaking and evasion of innate immunity.

### Cyclophilins at the Virus-Host Interface

The Cyp family of cellular proteins are key players at the virus-host interface. Cyps have peptidyl-prolyl isomerase (PPIase) activity and catalyze the *cis*/*trans* interconversion of the peptide bonds preceding proline (Davis et al., 2010). Thus, Cyps may modulate the structure or function of their target proteins through PPIase activity, or through modulating protein complex formation. Cyps are highly conserved in their PPIase domain, but differ in their subcellular localization (Hoffmann and Schiene-Fischer, 2014). For example, cyclophilin A (CypA) is cytosolic, while CypB is found in the endoplasmic reticulum (ER) (Nakagawa et al., 2005). CypA forms a complex with the classical immunosuppressant cyclosporine A (CsA), which leads to inhibition of calcineurin by the CsA-CypA complex and subsequent blockage of T-cell activation (Handschumacher et al., 1984; Takahashi et al., 1989). However, chemical modification of CsA allows the development of non-immunosuppressive cyclophilin inhibitors (CypI) that retain Cyp binding but do not inhibit calcineurin function. Non-immunosuppressive CsA derivatives, such as SCY-635 and alisporivir, have demonstrated clinical efficacy in the context of hepatitis C virus (HCV) infection (Sweeney et al., 2014).

**FIGURE 1:**
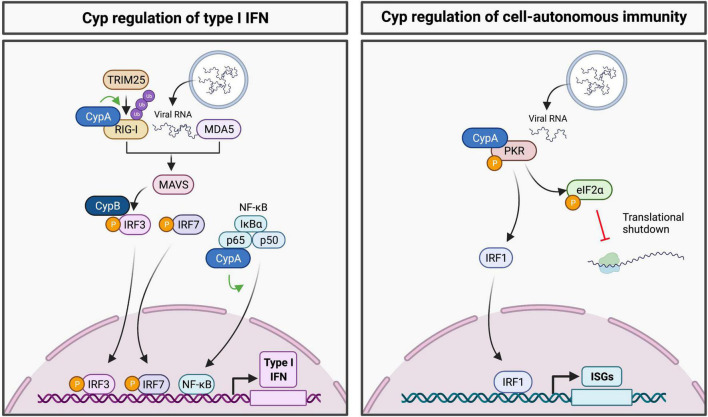
Regulation of innate immune signaling pathways by cyclophilins. **(Left panel)** Cyps have been shown to regulate RIG-I, IRF3 and NF-κB, thus contributing to activation of the type I IFN response. **(Right panel)** CypA regulates PKR activation to promote eIF2α phosphorylation and translational shutdown. Downstream of PKR, cyclophilin inhibitor treatment appears to activate transcription of ISGs in a PKR and IRF1-dependent manner.

Cellular cyclophilins are emerging as regulators of innate immune signaling pathways (Figure 1). For example, CypA regulates activity of the RNA sensor RIG-I by mediating its ubiquitination by the E3 ligase TRIM25 (Liu et al., 2017). Ubiquitination of RIG-I is necessary for its interaction with mitochondrial antiviral-signaling protein (MAVS), allowing engagement of further downstream antiviral signaling (Gack et al., 2007). Downstream of MAVS, CypB has been identified to regulate IRF3 phosphorylation and dimerization, where the interaction of CypB with IRF3 was shown to be necessary for the production of IFN-β in cells infected with Newcastle disease virus (Obata et al., 2005). Furthermore, CypA has been shown to interact with the p65 subunit of NF-κB to promote its stability and subsequent nuclear translocation of p65 (Sun et al., 2014). The RNA sensor PKR is also regulated by CypA, with CypA shown to interact with PKR and modulate its autophosphorylation (Daito et al., 2014). These findings emphasize the roles of Cyps at the virus-host interface.

CypA has been implicated in the replication of many RNA viruses, including HCV, dengue virus and other flaviviruses (Gallardo-Flores and Colpitts, 2021), coronaviruses (CoVs) and other nidoviruses (de Wilde et al., 2018), and HIV-1 (Liao et al., 2021). Numerous reports highlight the broad-spectrum antiviral activity of CypI. However, the roles of CypA in viral replication and the mechanisms underlying the broad-spectrum activity of CypI have remained elusive. To explain the broad requirement of viruses for Cyps as host factors, we propose that Cyps contribute to viral evasion of innate immunity. Given that Cyp-binding viral proteins such as HCV non-structural protein 5A (NS5A) are abundant in cells during active viral replication, it is possible that their interactions with Cyps may sequester CypA and CypB, limiting their ability to interact with and positively regulate innate immune signaling factors as described above. Sequestration of Cyps by these viral proteins may decrease antiviral immune signaling and contribute to viral immune evasion. Furthermore, recent findings suggest that multiple viruses may actively recruit CypA to aid in cloaking of their genomes from innate immune sensors. Here, we review the roles of CypA in viral immune evasion, focusing on viral cloaking as a unifying evasion mechanism.

## CypA and Viral Cloaking

### Positive-Sense RNA Viruses

A hallmark feature of positive-sense RNA virus replication is the rearrangement of host endomembranes into ROs (Romero-Brey et al., 2012). While ROs provide a platform for viral RNA replication (Paul et al., 2013), it is thought they also prevent the detection of replication intermediates by cytosolic PRRs (Neufeldt et al., 2016) and thus play a key role in viral evasion of antiviral innate immune signaling (Scutigliani and Kikkert, 2017). It has been proposed that disrupting RO formation or integrity may enhance antiviral immune signaling, in addition to directly impairing viral replication (Scutigliani and Kikkert, 2017).

**FIGURE 2:**
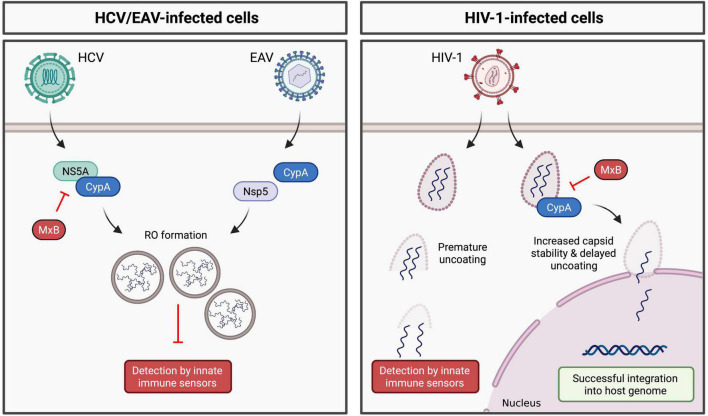
Regulation of viral cloaking by cyclophilin A. **(Left panel)** CypA modulates the ability of HCV and EAV to form membranous replication organelles, likely through direct or indirect interactions with HCV NS5A or EAV Nsp5, respectively. **(Right panel)** CypA enhances the stability of the HIV-1 capsid, thus prevent premature cytoplasmic uncoating. MxB inhibits HCV and HIV-1 replication in a CypA-dependent manner, likely disrupting viral cloaking.

Numerous studies have demonstrated a role for CypA in HCV replication (Chatterji et al., 2009). CypA binds to HCV NS5A (Foster et al., 2011; Madan et al., 2014), which plays a key role in formation of the double-membrane vesicles (DMVs) that comprise the HCV RO (Romero-Brey et al., 2012). In 2014, Madan et al. (2014) hypothesized that CypA promotes RO formation (and thus viral RNA cloaking) through its interaction with NS5A. Indeed, treatment with CypI (Chatterji et al., 2015; Colpitts et al., 2020) or silencing of CypA expression (Chatterji et al., 2015) significantly reduces the number and size of DMVs and concomitantly inhibits HCV replication. In CypA-silenced cells, DMV formation was rescued by addition of WT CypA, but not by a CypA H126Q mutant, which lacks PPIase activity (Chatterji et al., 2015). Importantly, RO formation in these studies was driven by exogenously expressed viral non-structural proteins (using an NS3-NS5B polyprotein expression system) and occurs independently of viral RNA replication, thus decoupling RO formation from viral replication. These findings suggest a specific role for CypA in mediating RO formation and thus HCV cloaking (Figure 2). CypA and NS5A interact directly *via* the proline-rich unstructured domain 2 of NS5A (Foster et al., 2011; Ngure et al., 2016). Notably, CsA treatment selects for the D316E/Y317N (DEYN) double mutation in domain 2 of NS5A. RO formation by the DEYN mutant is not inhibited by CypI treatment (Madan et al., 2014), suggesting that this mutation enables NS5A to induce RO formation independently of CypA. Collectively, these findings suggest that CypA promotes NS5A domain 2 structural rearrangements that mediate complex formation with NS5A interaction partners required for RO formation. Furthermore, HCV NS5A inhibits PKR activity (Gale et al., 1998), and CypA interacts with both NS5A (Hanoulle et al., 2009) and PKR (Daito et al., 2014), suggesting a model where CypA may regulate the ability of NS5A to antagonize PKR. Although the exact molecular mechanisms are not yet fully understood, CypA appears to regulate HCV NS5A-mediated immune evasion.

Like HCV, CypA is required for replication of equine arteritis virus (EAV) (de Wilde et al., 2013, 2018), a positive-sense RNA virus in the *Arteriviridae* family that similarly forms ROs comprised of DMVs (van Hemert et al., 2008; Knoops et al., 2012). Consistently, treatment with CsA inhibits EAV replication (de Wilde et al., 2013, 2018). EAV DMV formation depends on viral non-structural proteins (nsps) and cellular host factors (Snijder et al., 2001; de Wilde et al., 2013; van der Hoeven et al., 2016). Of note, EAV non-structural protein 5 (nsp5) has recently been implicated in modulating RO membrane curvature and DMV formation (van der Hoeven et al., 2016) and is thus critical for successful viral RNA synthesis (de Wilde et al., 2019). Interestingly, CsA treatment impairs formation of EAV DMVs (de Wilde et al., 2019), and CsA treatment selects for mutations in nsp5 within a potential CypA binding motif (de Wilde et al., 2019). These mutations render EAV DMV formation resistant to CsA treatment, reflecting the phenotype of the HCV DEYN mutant. Thus, as for HCV, CypA appears to regulate the ability of EAV to induce RO formation and cloak viral replication intermediates from innate immune sensors.

Like *Arteriviridae*, *Coronaviridae* belong to the Nidovirales order and also exhibit dependence on Cyps (de Wilde et al., 2018). While the functional roles of Cyps in CoV infection are still under investigation, it is worth noting that interactions between CypA and CoV nucleoprotein, nsp1 and nsp3 proteins have been identified (Luo et al., 2004; Pfefferle et al., 2011; Ma-Lauer et al., 2020). Given the roles of these CoV proteins in immune evasion and RO formation (Kasuga et al., 2021), it is possible that CypA may similarly contribute to CoV immune evasion. However, further studies are required to elucidate the functional roles of CypA in CoV infection and its potential contributions to CoV immune evasion.

### Human Immunodeficiency Virus

CypA has been shown to play conceptually similar roles in cloaking HIV-1 reverse transcription from innate immune detection. Successful HIV-1 infection relies on the viral capsid (CA) (Forshey et al., 2002; Rihn et al., 2013). CA regulates reverse transcription (Forshey et al., 2002) and enables encapsidated DNA synthesis (Jacques et al., 2016), as well as regulating nuclear import and integration of the viral genome (Matreyek and Engelman, 2011; Schaller et al., 2011). Furthermore, CA is critical for innate immune evasion (Lahaye et al., 2013; Rasaiyaah et al., 2013). Interactions with host factors regulate capsid stability and uncoating, thus contributing to viral replication and immune evasion, with CypA believed to play a critical role (Rasaiyaah et al., 2013). Binding of CypA to HIV-1 CA enhances viral replication in human cells (Ikeda et al., 2004; Song and Aiken, 2007). Consistently, viral replication is inhibited by mutating residues within the proline-rich CypA binding loop of CA to disrupt CypA-CA binding (Sokolskaja et al., 2004; Berthoux et al., 2005), or by treatment with CypI (Li et al., 2009; Rasaiyaah et al., 2013), implicating CypA as a critical pro-viral binding partner of the viral CA. More recently, an additional CypA binding site was identified, in which CypA bridges CA molecules within adjacent hexamers, stabilizing the CA lattice structure (Liu et al., 2016). It is thought that CypA regulates CA stability to prevent pre-mature uncoating in the cytoplasm, thus helping to cloak reverse-transcribed viral DNA from cytoplasmic DNA sensors (Figure 2).

Interestingly, infection with HIV-1 CA mutants deficient in CypA binding induces a type I IFN response in infected cells, suggesting that CypA binding is crucial for CA integrity and cloaking of reverse-transcribed viral DNA (Rasaiyaah et al., 2013). The P90A HIV-1 CA mutant, which is unable to interact with CypA, showed increased IFN-β production compared to WT HIV-1, as well as decreased virus replication (Rasaiyaah et al., 2013). Indeed, several studies have suggested that HIV-1 requires CypA to cloak its genetic material within the cytoplasm and prevent the activation of innate immune sensors and antiviral signaling cascades in primary human macrophages and dendritic cells (Li et al., 2009; Manel et al., 2010; Rasaiyaah et al., 2013; Hilditch and Towers, 2014). Furthermore, the CA-CypA interaction protects HIV from intrinsic viral restriction, such as that mediated by TRIM5α (Kim et al., 2019). Disruption of CA-CypA binding *via* mutation leads to premature uncoating by accelerating capsid disassembly, and ultimately reducing viral infectivity (Li et al., 2009). Importantly, premature uncoating induced by impaired CA-CypA binding is thought to uncloak newly synthesized viral DNA, resulting in its detection by intracellular innate sensors (Rasaiyaah et al., 2013). Overall, CA-CypA binding stabilizes the HIV-1 capsid core and prevents premature uncoating, ultimately promoting reverse transcription and evasion of innate immune sensors and restriction factors. Interestingly, a similar role was identified for CypA in regulating uncoating of enterovirus-71 (Qing et al., 2014), although induction of antiviral immune responses was not assessed in this study.

## Disruption of CypA-Mediated Viral Evasion

### Cellular Antiviral Effector Mechanisms Targeting CypA Dependence

CypA-dependent viral cloaking may be targeted by cellular antiviral effector proteins, particularly the IFN-inducible myxovirus resistance B (MxB) protein, which exerts broad antiviral activity. Yi et al. (2019) showed that the inhibition of HCV replication by MxB is dependent on CypA. Notably, MxB and CypA both bind NS5A, where MxB binding to NS5A domain 1 prevents CypA interaction with domains 2 and 3 (Yi et al., 2019). The binding of MxB to NS5A also prevents its localization to the ER (Yi et al., 2019), which together with disruption of CypA-NS5A binding may hinder the formation of the HCV RO and therefore disrupt HCV cloaking (Figure 2). MxB also exerts antiviral activity against other *Flaviviridae*, such as Japanese encephalitis virus (JEV) and dengue virus (DENV) (Yi et al., 2019), which depend on Cyps for their replication, as was recently reviewed (Gallardo-Flores and Colpitts, 2021). Interestingly, CypB interacts with JEV NS4A (Kambara et al., 2011), which mediates membrane rearrangements forming the flavivirus RO, while CypA may also contribute to formation of the flavivirus replication complex (Qing et al., 2009). Therefore, it is possible that, as for HCV, MxB may similarly disrupt Cyp-mediated cloaking of flaviviruses, although future studies are needed to clarify the roles of Cyps in flavivirus replication and cloaking. Furthermore, future studies will need to address whether MxB antagonism leads to an increase in IFN production, as would be expected if the mechanism involves disruption of viral cloaking.

MxB exerts similar CypA-dependent antiviral activity in the context of HIV-1 infection, where silencing of CypA expression dampens MxB inhibition of HIV-1 (Liu et al., 2013) and addition of CsA rescues HIV-1 from MxB restriction (Miles et al., 2020). Since the effect of CsA and MxB is not additive, one possibility is that MxB and CypI restrict HIV-1 replication *via* a shared mechanism involving disruption of CA-CypA cloaking. The dependence of MxB restriction on CypA was further highlighted by studies showing that mutations in the CypA binding loop of HIV-1 CA confer resistance to MxB restriction (Liu et al., 2015; Miles et al., 2020). Collectively, these findings show that CypA recruitment by CA is important for MxB restriction, suggesting that MxB may disrupt HIV-1 CypA-dependent cloaking mechanisms. However, whether MxB restriction concomitantly leads to an increase in IFN production, as would be expected if cloaking is disrupted, is still unknown.

### Cyclophilin Inhibitors and Antiviral Immunity

Recent studies suggest that CypI bolster antiviral signaling pathways, which may contribute to the broad-spectrum antiviral activities of CypI. In the context of HCV, CypI treatment blocks the formation of ROs (Madan et al., 2014; Chatterji et al., 2015; Colpitts et al., 2020), thereby disrupting viral cloaking. Consistently, Liu et al. (2011) demonstrated that CsA restored IFN-α mRNA levels in HCV-infected hepatocytes. The relevance of CypI-induced IFN-α signaling was confirmed in a clinical trial evaluating the efficacy of the CypI SCY-635 in patients chronically infected with HCV (genotype 1). Notably, HCV patients treated with SCY-635 displayed increased plasma concentration levels of IFN-α, coupled with reduced viral load (Hopkins et al., 2012). Additionally, SCY-635 treatment increased plasma concentrations of IFN-λ as well as OAS-1, a known ISG (Hopkins et al., 2012). Interestingly, CsA treatment mildly induced IRF1 expression in HCV-infected Huh7 cells (Liu et al., 2011) and enhanced the expression of IRF1-dependent ISGs in HCV-replicating Huh7 cells, including *CCL2*, *MX1*, *RSAD2* and *IFIT2*, as well as *IFN-*β (Colpitts et al., 2020). These findings are consistent with disruption of HCV cloaking or other Cyp-dependent viral evasion mechanism by CypI treatment, resulting in restored antiviral immune signaling.

Li et al. (2018) reported a synergistic effect of CsA in promoting IFN-α signaling against Middle East respiratory syndrome coronavirus (MERS-CoV). PCR array revealed that combined treatment of *ex vivo* lung samples with IFN-α and CsA increased the induction of ISGs compared to IFN-α or CsA treatment alone (Li et al., 2018). Following CsA treatment of MERS-CoV-infected cells, IFN-λ protein levels were upregulated, although IRF1 and ISG levels were not evaluated (Sauerhering et al., 2020). Interestingly, silencing of IRF1 or blockade of IFN-λ abrogated CypI inhibition of MERS-CoV infection (Sauerhering et al., 2020), reflecting similar findings where the potency of CypI against HCV infection depended on IRF1 expression (Colpitts et al., 2020). Interestingly, CsA treatment of uninfected Calu-3 lung cells induced expression of IRF1, IFN-λ, and antiviral ISGs, including *MX1*, *OAS1*, *IFIT2*, *BST2*, and *RSAD2* (Sauerhering et al., 2020), suggesting that CsA treatment itself contributes to antiviral signaling. Further studies are required to evaluate the mechanisms underlying this observed induction of ISGs following CsA treatment.

Similar effects have been observed in the context of HIV-1 infection. Treatment with SmBz-CsA, a non-immunosuppressive CsA analog, enhanced IFN-β mRNA production in HIV-1-infected human monocyte-derived macrophages (Rasaiyaah et al., 2013). This phenotype was attributed to CypI disruption of the CypA-CA interaction, resulting in uncloaking and premature uncoating leading to innate immune recognition of HIV-1 DNA in the cytoplasm. In addition to highlighting a key role for the CypA-CA interaction in evading innate immune recognition, these findings were the first to suggest a novel antiviral mechanism for CypI based on uncloaking of viral PAMPs and engagement of antiviral innate immunity.

Notably, CsA treatment has been shown to enhance IFN signaling during other viral infections. In the context of rotavirus (RV; a double-stranded RNA virus), IFN-β mRNA was increased following CsA treatment of rotavirus-infected cells (Shen et al., 2013). CsA induction of IFN-β was also demonstrated *in vivo*, where CsA treatment of RV-infected neonatal mice upregulated IFN-β levels in the intestine as well as the spleen (Shen et al., 2015). While CsA treatment seems to promote IFN signaling and reduce RV replication, it is interesting to note that He et al. showed in 2012 that CypA regulates type I IFN production. CypA overexpression inhibited RV titers and promoted IFN-β production, while CypA silencing enhanced viral replication and diminished IFN-β production (He et al., 2012), consistent with the positive regulation of innate immune signaling factors by CypA described above. Intriguingly, a similar effect is seen in influenza A virus (IAV), where both CypA (Liu et al., 2012b; Li et al., 2016) and CsA (Liu et al., 2012a) inhibit IAV replication. It is possible that CsA treatment disrupts viral cloaking or sequestration of Cyps by viral proteins, thus restoring sensing pathways that detect viral RNA or DNA in the cytoplasm of infected cells. However, further studies are required to elucidate the underlying mechanisms.

## Discussion

Multiple unrelated viruses appear to have evolved CypA dependence for cloaking of viral genomes or viral replication intermediates in the cytoplasm of infected cells. It is likely that CypI treatment disrupts these viral cloaking mechanisms, exposing viral replication intermediates to innate immune sensors and restoring antiviral immune signaling. Furthermore, the regulation of RIG-I, NF-κB and PKR by CypA highlights its roles in potentiating innate immune signaling. Through interactions with CypA, viral proteins may sequester CypA away from innate immune signaling pathways, thus preventing the induction of downstream IFN signaling. Upon CypI treatment, the interaction between viral proteins and CypA may be disrupted, allowing CypA to interact with host innate sensors, perhaps using an alternate binding site. Notably, CypI treatment has been shown to promote an antiviral state in cells, likely through a combination of disruption of viral cloaking and regulation of innate immune signaling pathways. While further studies are required to understand the underlying mechanisms, the findings reviewed here highlight the roles of CypA in viral cloaking and provide a potential mechanism to explain the broad-spectrum antiviral activity of CypI.

## Author Contributions

CC conceptualized and supervised the review. JM, IP-D, and CG-F conducted the literature review and wrote the first draft. JM and IP-D designed the figures. JM and CC edited and revised the manuscript. All authors have read and agreed to the published version of the manuscript.

## Conflict of Interest

The authors declare that the research was conducted in the absence of any commercial or financial relationships that could be construed as a potential conflict of interest.

## Publisher’s Note

All claims expressed in this article are solely those of the authors and do not necessarily represent those of their affiliated organizations, or those of the publisher, the editors and the reviewers. Any product that may be evaluated in this article, or claim that may be made by its manufacturer, is not guaranteed or endorsed by the publisher.
